# Supplementary material for: Operational thresholds of urease-mediated microbial cementation: Multivariate optimization and field validation in ambient groundwater environments
Source: PLoS One. 2025 Aug 22;20(8):e0330481. doi: 10.1371/journal.pone.0330481 (PMC12373166; doi:10.1371/journal.pone.0330481)
Supplement: S1 Table — Includes data from abiotic control, biological control, and NBPT-MICP groups. Values are presented as mean ± standard deviation. (DOCX) [file pone.0330481.s001.docx]

**Statistics of the test results of the samples**

| **Parameter** | **Abiotic Controls** | **Biological Controls** | **NBPT-MICP (0.1%)** |
| --- | --- | --- | --- |
| **UCS (MPa)** | 0.022 ± 0.003 | 2.68 ± 0.21 | 2.53 ± 0.18 |
| **CaCO₃ content (wt%)** | 0.11 ± 0.03 | 12.34 ± 1.87 | 11.86 ± 1.15 |
| **Sectional CV (%)** | N/A | 28.7 | 9.7 |
| **Permeability (m/s)** | 2.08 × 10⁻³ | 6.9 × 10⁻⁵ | 6.4 × 10⁻⁵ |
