# Supplementary material for: Operational thresholds of urease-mediated microbial cementation: Multivariate optimization and field validation in ambient groundwater environments
Source: PLoS One. 2025 Aug 22;20(8):e0330481. doi: 10.1371/journal.pone.0330481 (PMC12373166; doi:10.1371/journal.pone.0330481)
Supplement: S2 Table — Shows conversion rates, standard deviations, and significance levels for comparisons between groups with varying Ca2⁺ to urea ratios. (DOCX) [file pone.0330481.s002.docx]

**Statistical data of BCR test results**

| **BCR** | **Conversion Rate (with NBPT) (%)** | **Standard Deviation** | **Statistical Significance (vs. BCR=2:1)** |
| --- | --- | --- | --- |
| 3:1 | 104.0 | 0.9 | p=0.78 |
| 2:1 | 104.2 | 1.0 | Reference |
| 1:1 | 103.9 | 0.8 | p=0.42 |
| 1:2 | 97.3 | 1.2 | p<0.01 |
| 1:3 | 89.0 | 1.5 | p<0.001 |
